# Leveraging State-of-the-Art AI Algorithms in Personalized Oncology: From Transcriptomics to Treatment

**DOI:** 10.3390/diagnostics14192174

**Published:** 2024-09-29

**Authors:** Anwar Shams

**Affiliations:** 1 Department of Pharmacology, College of Medicine, Taif University, P.O. Box 11099, Taif 21944, Saudi Arabia; a.shams@tu.edu.sa or anwar.shams@mail.mcgill.ca; Tel.: +00966-548638099; 2 Research Center for Health Sciences, Deanship of Graduate Studies and Scientific Research, Taif University, Taif 26432, Saudi Arabia; 3 High Altitude Research Center, Taif University, P.O. Box 11099, Taif 21944, Saudi Arabia

**Keywords:** artificial intelligence, deep learning, genome transcriptomic, prediction, therapeutics, personalized oncology

## Abstract

Background: Continuous breakthroughs in computational algorithms have positioned AI-based models as some of the most sophisticated technologies in the healthcare system. AI shows dynamic contributions in advancing various medical fields involving data interpretation and monitoring, imaging screening and diagnosis, and treatment response and survival prediction. Despite advances in clinical oncology, more effort must be employed to tailor therapeutic plans based on each patient’s unique transcriptomic profile within the precision/personalized oncology frame. Furthermore, the standard analysis method is not compatible with the comprehensive deciphering of significant data streams, thus precluding the prediction of accurate treatment options. Methodology: We proposed a novel approach that includes obtaining different tumour tissues and preparing RNA samples for comprehensive transcriptomic interpretation using specifically trained, programmed, and optimized AI-based models for extracting large data volumes, refining, and analyzing them. Next, the transcriptomic results will be scanned against an expansive drug library to predict the response of each target to the tested drugs. The obtained target-drug combination/s will be then validated using in vitro and in vivo experimental models. Finally, the best treatment combination option/s will be introduced to the patient. We also provided a comprehensive review discussing AI models’ recent innovations and implementations to aid in molecular diagnosis and treatment planning. Results: The expected transcriptomic analysis generated by the AI-based algorithms will provide an inclusive genomic profile for each patient, containing statistical and bioinformatics analyses, identification of the dysregulated pathways, detection of the targeted genes, and recognition of molecular biomarkers. Subjecting these results to the prediction and pairing AI-based processes will result in statistical graphs presenting each target’s likely response rate to various treatment options. Different in vitro and in vivo investigations will further validate the selection of the target drug/s pairs. Conclusions: Leveraging AI models will provide more rigorous manipulation of large-scale datasets on specific cancer care paths. Such a strategy would shape treatment according to each patient’s demand, thus fortifying the avenue of personalized/precision medicine. Undoubtedly, this will assist in improving the oncology domain and alleviate the burden of clinicians in the coming decade.

## 1. Introduction

Cancer continues to be a global health burden, showing ascending demographic incidence, with 420 million new cancer cases anticipated to emerge by 2025 [1,2,3,4]. Worldwide, cancers of the female breast, prostate, lungs and bronchi, and colon and rectum represent the topmost cancer prevalence [2,3]. Additionally, lung and bronchus cancers are predominating as the leading cause of cancer-related death among both sexes [2,3]. Like other medical fields, the goal of therapy in oncology is to maximize cancer control and reduce therapeutic toxicity, thus improving the quantity and quality of the patient’s life [5]. Cancer treatments can be classified into conventional (traditional) and modern approaches [1]. Conventional paradigms comprise surgery, chemotherapy, radiation therapy, or a combination plan [6]. With the recent advances in cancer treatment, innovative methods that should overwhelm the drawbacks of traditional therapies have also been performed, including targeted therapy, stem cell therapy, gene therapy, ablation therapy, nanoparticle therapy, natural antioxidants, and ferroptosis-based therapy [1,7]. Furthermore, radionuclides have displayed fundamental elements that evolved in radiopharmaceutical research, with the drive to be used in cancer diagnosis and treatment [8]. Radionuclides are unstable particles that emit specific types of radiation and can label biomolecules or drugs for diagnostic purposes, such as positron emission tomography (PET) and/or single-photon emission computed tomography (SPECT) [8]. Also, preclinical radiopharmaceutical research has validated radionuclides’ cytotoxic and genotoxic effects by emitting ionizing radiation after being absorbed by the cancer cells and leaving the normal tissues unaffected [9,10]. Yumiko et al. examined the anticancer effects of the combination of doxorubicin and [99mTc]MIBI chemoradiotherapy in non-small-cell lung cancer [11]. In vitro accumulation of the [99mTc]MIBI in human lung cancer cells resulted in increased sensitivity to doxorubicin cytotoxic effects in irradiated tumours in the xenograft mice [11].

However, several ongoing struggles remain associated with cancer treatment, involving drug resistance, either primary or acquired, relapse and metastasis, lack of specificity, and detrimental adverse effects [1]. Among these pitfalls, tumour heterogeneity/genomic and molecular alteration constitute the most challenging concern, hindering the selection of the druggable regimen and limiting the attainment of precision oncology [1]. Therefore, applying the precise genetic picture for each patient to develop a remedy that targets given molecular abnormalities is warranted. In this realm, the arrival of tumour molecular profiling technologies such as next-generation sequencing (NGS) to predict targetable molecular biomarkers has materialized a revolution in the personalized/precision cancer medicine landscape [12,13]. These transcriptomic technologies can play a significant role in molecular pre-screening in clinical research to determine the dynamic expression of each transcript [12,14,15]. In this regard, RNA sequencing is a powerful tool used to distinguish differentially expressed genes, isoforms, and transcripts by producing less noisy and more normalized data analysis [15]. Hitherto, the relatively high error rates during clinical interpretation of such large amounts of data and high sequencing costs have limited their clinical implementation [14,16,17].

Recently, the substantial contribution of artificial intelligence (AI) in developing different aspects of the biomedical field, including basic and clinical oncology, has been significantly increasing [18,19]. The advent of the deep learning model (DL) has delivered high-power capacity to extract and integrate enormous amounts of complicated data such as genomic sequencing analysis [19,20]. DL is an emerging subfield of AI that has arisen in the field to overcome the limitations of the primary AI version, machine learning (ML) [15,21]. DL uses raw data to generate a scientific conclusion called automated feature engineering [15,21]. This characteristic allows DL to be engaged in various clinical applications ranging from early cancer diagnosis, grading and staging, molecular identification of tumours, prediction of treatment response and patient prognosis, precision treatment, new drug discovery, and designing clinical trials [19,20]. Currently, AI technologies are acknowledged to have some clinical uses, mainly in radiology [22], endoscopy, dermatology, and pathology, encompassing early imaging screening and diagnosis, detection of different pathological biomarkers, and optimized selection of therapeutic plans [20,23,24,25,26,27]. A valuable example of this application is the ability of AI to discriminate targeted epidermal growth factor receptor (EGFR) mutations from the wild type in non-small-cell lung cancer (NSCLC) by using the PET/CT-based DL model, allowing for accurate patient selection and the delivery of appropriate therapy [26,28]. The expansion of AI in medicine is gaining momentum, and numerous technical innovations to improve AI eminence have been employed since 2022 [24]. 

In oncology, the characterization of the genomic background of each patient and the identification of the association of each mutation with clinical phenotypes, patient prognosis, and effective treatment is a bottleneck for cancer genomic medicine [18]. This is attributed to the complexity of RNA gene expression data and duplication values, mandating a precise approach to data dissection and analysis [15]. Furthermore, the response to treatment differs vastly from one patient to another, resulting in the resurgence of individualized/personalized care for each patient [5]. Personalized medicine denotes the tailoring of treatment for each patient, relying on specific genomic aberrations. Hence, it ensures therapeutic benefits for those who are more likely to respond and spares expense and adverse effects for those who will not [29]. Therefore, a need for potent tools for extracting and analyzing high-dimensional records exists that can be addressed efficiently through ML and DL algorithms [15]. Given the exponential growth of accessible molecular figures and genomic data points, DL-based algorithms (particularly the CNN model) have demonstrated a comprehensive avenue with great accuracy in determining the tumour molecular phenotype and the transcriptomic profile, and scoring the expression of specific tumour marker proteins [15,18,27]. Moreover, through the automatic integration of medical data, DL algorithms can build models to predict the risk of tumour recurrence and patients’ response to treatments, thus guiding physicians in making precise therapeutic interventions [24,29]. For example, integrating DL models using radiomics, i.e., extracting numerical data from imaging [30], and pathomics, i.e., extracting and mining quantitive data from digitized histopathology imaging [31], has allowed researchers to predict biomarkers associated with immunotherapy response in aggressive melanoma with a high accuracy rate [32]. 

Consequently, leveraging AI in clinical oncology will illuminate current issues and assist in driving meaningful investigations and therapeutic strategies that will ultimately advance the field of clinical practice [5]. Many challenges must be met to achieve fruitful patient outcomes, particularly intratumour heterogeneity, comprehensive analysis of molecular genomic data, and finding a druggable molecular pathway/target within the framework of personalized treatment. Within this framework, a recent prestigious study was conducted in Italy among 486 breast cancer patients enrolled at IRCCS Istituto Tumori [33]. The explainable artificial intelligence (XAI) integrated model has been employed to predict the probabilities of 5-year and 10-year events associated with invasive breast cancer and to select the therapeutic plan for each patient. These events include numerous features such as the type of surgery, chemotherapy scheme, age, tumour diameter, lymph node status (involvement, eradication, and dissection), hormonal receptors status (ER and PgR), and Ki67 grade [33]. The application of this system potentiates the interplay between medical artificial intelligence and clinical practice, guiding and fortifying clinical decisions within the context of personalized medicine [33]. 

The goal of our study is to optimize the choice of treatment strategy for individual cancer patients by overcoming the current pitfalls. This will be achieved by comprehensively revising current AI-based knowledge and describing a novel cancer diagnosis and treatment proposal for each cancer patient. Hence, we propose employing AI-based technology (such as DL-related algorithms) during clinical investigations of cancer patient candidates to analyze the outcomes of the transcriptomic modality (e.g., RNA/NGS sequencing). This will provide broad elucidation of genetic graphing for each patient and map a clear picture of all ongoing aberrations involving the identification of molecular pathway alterations and gene dysregulations, as well as the detection of multiple druggable targets. Next, the obtained transcriptomic results should be paired with the available drug libraries (including Food and Drugs Administration (FDA)-approved or investigational drugs) to predict the best therapeutic option based on each patient’s unique genetic figure. Consequently, the AI system would provide a target–drug combination by computationally identifying responding patients from non-responders or, in other words, predicting the response percentage to each given agent. This concept will potentially establish a unifying dynamic multitask model that enables profound interpretation of large amounts of omics data and simultaneously recommends the best therapeutic combinations. To the best of our knowledge, this work would offer a holistic approach for healthcare providers to refine therapeutic plans according to each patient’s needs. Ultimately, it can introduce a novel technology that guarantees rapid and precise decisions and reduces the need for preclinical laboratory experimental validation, providing a cost-effective and time-saving approach.

## 2. Methodology and Technical Procedures

1. Target population:

The present strategy will be applied to critically ill cancer patients who have been diagnosed with an aggressive phenotype, have a strong family history of a given cancer, or have developed a recurrent disease where the standard therapeutic regimen showed a failure to achieve complete remission.

2. The components of the proposed model:

We are suggesting several foundation steps of supervised AI-learning models to facilitate the implantation process, including the following:

### 2.1. Data Collection and Preprocessing

The initial step is to collect genetic sequencing data extensively from valid resources such as scientific reports and patient records [34]. To extract the most relevant literature, the identification of differentially expressed genes and the analytical knowledge of various tumour types and corresponding therapeutic modalities must be addressed [15,35,36]. For this purpose, many available online databases have been suggested to collect the relevant information regarding cytopathology, histopathology, and the underlying genetic architecture of different disorders, including Online Mendelian Inheritance in Man (OMIM) [37] and the Pathologisch Anatomisch Landelijk Geautomatiseerd Archief (PALGA) [38]. Similarly, numerous public repositories have specialized in providing demarcated clinical genetic analysis in diverse aspects, such as the Gene Expression Omnibus (GEO) [39], the Cancer Genome Atlas (TCGA) [40], the Cancer Cell Line Encyclopedia (CCLE) [41], the ENCyclopedia Of DNA Elements (ENCODE) [42], and the Catalogue of Somatic Mutations in Cancer (COSMIC) [43]. Also, DrugBank [44], the Therapeutic Targets Database (TTD) [45], PubChem [46], and ChEMBL [47] are examples of web-enabled databases containing broad molecular information about drugs, their chemical structures, mechanisms of action, interactions, side effects, and their potential targets [36]. These data should then be subjected to systematic and exhaustive revision to maintain good quality and extract noise and irrelevant information.

### 2.2. Model Selection 

Different frameworks and tools for AI-related models have been developed. They can be joined to augment the efficiency and precision of the genome sequencing (e.g., RNA-seq/NGS) interpretation, resulting in deeper insight and solid conclusions in various biological and clinical research fields [15,18,27,48,49]. AI-ML models have been proposed to classify the dataset for different cancer types. These include random forest (RF), support vector machine (SVM), K nearest neighbour (KNN), neural network (NN), and gradient boosting machine (GBM) [50]. Among these models, RF was the most efficient and reliable model in cancer classification compared to other approaches [15,50]. The current study recommends employing sophisticated DL algorithms, specifically deep neural networks, designed to manage complicated relationships within multifaceted genetic data [51]. DL modality uses multilayer neurons to handle high-level constructs that show the differences between samples. The power of DL is attributed to its ability to mine complicated data on links or nodes and build biological relationship networks among genes to discover a druggable target [52].

Moreover, various DL-based approaches have been deployed to extract, denoise, analyze, and classify differentially expressed genes from RNA sequencing/transcriptomic datasets of different types of cancers [15]. These involve profound deep learning model algorithms such as convolutional neural networks (CNNs), recurrent neural networks (RNNs), and natural language processing (NLP) [15,18,27,48,49,53,54]. DL algorithms (CNNs and RNNs) can be used to categorize various types of RNA data, such as coding and non-coding RNAs [53], interpret gene expression data from RNA-seq experiments, and recognize patterns and relationships that might be challenging to distinguish through standard statistical methods [5,14,15,48,49]. Furthermore, Xiao et al. proposed a DL-based multimodal ensemble system composed of five ML algorithms: SVM, RF, DT (decision trees), KNN, and GBDT (gradient-boosting decision trees) [55]. This multimodal system demonstrated high accuracy in classifying different types of cancers, specifically lung, breast, and stomach cancer [15,55]. Likewise, this AI-based technology can further show the aberrantly regulated pathways (down-regulated vs. up-regulated) during cancer progression compared to standard cells [15,48,49]. Also, functioning AI algorithms allow the detection of RNA sequence variants, including single nucleotide polymorphisms (SNPs) and insertions/deletions (indels), prediction of alternative splicing events, recognition of secondary and tertiary RNA structures, and identification of different biomarkers associated with specific diseases [56]. While both CNN and RNN tools have been combined to analyze extensively RNA sequencing data, the DL-NLP algorithm is widely used to extract and refine relevant information and databases from a broad scientific literature and incorporate it with RNA sequencing data obtained using CNNs and RNNs [15,18,48,49,57,58]. Regarding CNN architecture, a novel model combining a CNN and a binary particle swarm optimization decision tree (BPSO—DT) has been suggested to precisely extract relevant features and classify multiple cancers, including renal cancer, breast cancer, lung cancer, and uterine cancers [49].

### 2.3. Training the Model 

We suggest that a supervised learning technique be applied to the proposed model. The training step has two phases:

Phase one: The proposed program will be supported with all available public genome-wide sequence-related scientific literature that has been experimentally investigated and validated. This is followed by training the system on data extraction and cleaning examples with known outcomes. 

Phase two: The intended program will be supplemented with a broad drug library containing all available FDA-approved anticancer drugs and investigational drugs in clinical trials. This is followed by training the model on a large dataset of known drug responses with their corresponding RNA sequences.

### 2.4. Interpretability of the Model 

Interpretability, on its own, is a general concept defined as the extraction of specific types of information. This can span from planning an experiment to envisioning its final results [59]. Under the umbrella of AI models, interpretability relates to generating insightful data from ML algorithms by extracting relevant information while considering the interplay relationships that are either contained in the original data or trained into the model. This interpretable ML model can also be called explainable ML, intelligible ML, or transparent ML [59]. Introducing interpretability features during the design of an AI model is vital to determining poor vs. good performance and accessing the quality of input/outcome data that can be useful in model building and implementation [60]. In clinical oncology, this tool is crucial to an accurate decision-making process [61]. Ennab et al. designed an interpretable AI-based model incorporated with a system mirroring human abilities in interpreting statistical data [62]. This was achieved by measuring the weight of the relative parameters extracted from medical images and the patient’s clinical symptoms. This technology was also deployed to navigate different possibilities for the occurrence of the disease, applying statistics and probability rules [62]. In our proposed model, we aim to boost the interpretability concept and the transparency of the decision-making process by adopting the following procedures [62]:

- Feeding the model with insightful, relevant information that can be produced in various formats, such as natural language, visualization, or mathematical equations with meaningful relationships.
- Training the model to deliver clear decisions and explanations for the results.
- Programming the model to provide the likelihood of positive and negative values for each specific sample.

Reducing the number of complex steps that can offset the model’s interpretability. Consequently, this will represent significant progress in demonstrating the resulting decisions in medical diagnosis and providing a transparent picture of each medical scenario. 

### 2.5. Feasibility of the Model 

The feasibility analysis of AI-based models in medical oncology depends on evaluating several aspects, including data quality, practical application, utility, ethical and regulatory approval, cost-effectiveness, and incorporation into clinical workflow on cancer diagnosis and treatment [63]. On the other spectrum, several hurdles related to human experience, bias, technical considerations, and data security must be offset [63]. For effective widespread implementation, the AI-based algorithm must fully address the following issues:

#### 2.5.1. Clinical Utility

AI systems have shown promising results in cancer diagnosis and screening by precisely analyzing radiological and pathological figures with comparable performance to human expertise [63]. The best example of this application is using AI technology in the early diagnosis of NSCLC [64,65]. Additionally, AI models can predict patients’ prognoses and tailor their therapeutic plans by estimating possible complications depending on several types of clinical information, such as the patient’s history, cancer genomics, and treatment responses [66]. 

#### 2.5.2. Data Availability and Quality

One big challenge in data availability is acquiring comprehensive and standardized datasets from different health institutions due to patients’ privacy and variability in clinical practice. AI models use high-quality datasets to deliver accurate results, but incomplete or inconsistent data gathering can degrade an AI system’s performance [67]. 

#### 2.5.3. Technical Feasibility

Modified versions of AI models, such as the DL algorithm, can adapt and interpret the big complex tasks associated with clinical oncology, such as the radiological identification of tumours [68]. However, routine optimization and updating of the model is necessary to maintain trustable results. Moreover, introducing AI models into clinical practice requires robust informational technology (IT), infrastructure encompassing data storage, highly secure systems, and precise computing [68]. 

#### 2.5.4. Regulatory and Ethical Considerations

For AI models to be used in clinical practice, routine safety assessment and regulatory approval from the FDA are required [63]. Furthermore, ethical validation and patient consent should also be obtained to ensure transparency and security of the whole process. This process can be expensive and time consuming, which might add another hindrance during AI implementation and feasibility [63].

#### 2.5.5. Cost-Effectiveness

This includes the implementation process and the investment results. While introducing AI models in clinical frameworks can eventually reduce the cost burden by improving the accuracy of different investigational tools, the employment of this technology itself demands significant investments and training [69]. However, long-term applications can lower overall costs by providing a better therapeutic plan for each patient and thus reducing hospital stays and requirements [70].

#### 2.5.6. Clinical Integration

Common obstacles experienced during AI model integration into healthcare systems are resistance to adopting AI technology, lack of training, and disquiet about increasing current workloads [70,71]. Indeed, AI systems complement human experience, and the process should be introduced in such a way that AI works as an assistant in optimizing final decisions while maintaining clinician oversight [71].

#### 2.5.7. Pilot Studies and Real-World Applications

Numerous studies have investigated and validated the feasibility of different AI models in real-world applications [64,65,72,73]. A study by Diao et al., conducted in China using a mobile CT scanner, demonstrated the diagnostic efficacy of an AI model in detecting high-risk pulmonary lesions with a significant reduction in diagnostic time. Thus, implementing such a model as a second reader would avoid missed diagnoses and potentiate the radiologists’ confidence [72]. Similarly, AI has brought significant innovations in neuro-oncology, specifically in brain glioma diagnosis, categorization, treatment planning, and prognosis prediction [73]. A modified three-dimensional CNN model with 11 layers was examined to detect challenging brain lesions, including brain injuries, tumours, and ischemic lesions. This model proved its efficiency and enabled its implementation in various clinical settings [74]. Moreover, combining an ML algorithm with a bioinformatics platform resulted in a new major histocompatibility complex (MHC) recognition tool, identifying an essential molecule in infection, autoimmunity, transplantation, and cancer immunotherapy. This technology assisted in the classification of MHC I and MHC II with an accuracy reaching 91.66%, and an online website was built for this purpose, with the following URL: http://lab.malab.cn/~acy/BioseqData/Protein.html, accessed on 6 August 2024 [75].

### 2.6. Optimization of the Model 

This step is crucial after training the model to ensure the accuracy of the embedded data and improve the system’s performance. The model is adjusted by fine-tuning the parameters and refining its inner structures. Continuous optimization and follow-up processes should be established to continue updating the inserted data loop as our knowledge of genetic materials grows. In this regard, the moth–flame optimizer system (MFO) is an algorithm belonging to the swarm intelligence family that has been used widely to increase the speed and accuracy of a designed model [76,77]. This system can solve various numerical problems, multilevel image segmentation parameters, and clinical cases with a maximum performance capacity, making it the most feasible technique for resolving optimization settings [76]. MFO algorithms have been deployed successfully to manage the optimization problem in numerous sectors, such as image processing, power and energy systems, engineering design, and medical applications [77].

### 2.7. Integration into Healthcare Systems 

**Figure 1:**
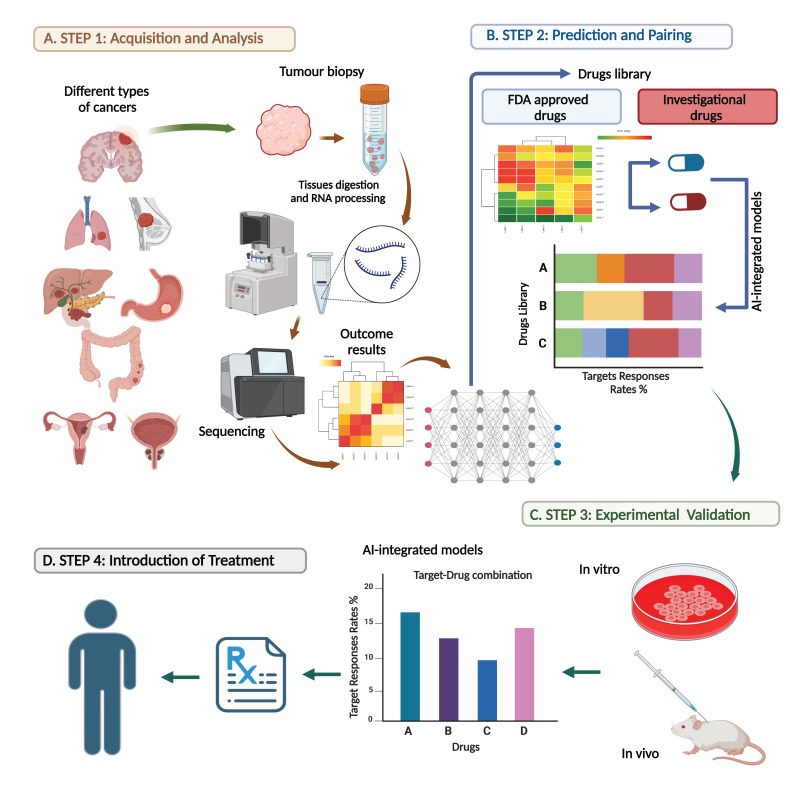
Proposed methodology diagram: An overview of integrating the transcriptomic technology and AI models into clinical oncology, aiding the diagnosis and therapeutic decision process. (**A**) Tissue samples collected from different types of cancers will then be processed for tissue digestion and RNA extraction, preparing samples for comprehensive transcriptomic analysis using AI-based technology. (**B**) The transcriptomic results will be examined against an extensive drug library (both FDA-approved and investigational) to predict the response of each target to the tested drugs (presented as Targets Responses Rates%) producing target–drug combinations. (**C**) The obtained target–drug pairs will be validated experimentally in in vitro and in vivo models. (**D**) Finally, the best treatment options will be selected and discussed with the patient before the introduction of a therapy. (Created with BioRender.com, https://app.biorender.com/gallery, accessed on 6 August 2024).

The settled AI models are then incorporated into the healthcare system to guide physicians in making precision diagnoses and therapeutic decisions, as demonstrated in Figure 1.

#### 2.7.1. Acquisition and Analysis Step

After operating on the cancer, a tissue biopsy will be obtained and sent for standard immunohistochemistry analysis to determine the clinical staging and grading of the tumour. Additionally, part of the tumour biopsy will also be subjected to transcriptomic exploration after experimental processing of the obtained tumour. Because the naïve pooling and averaging statistical analysis for genome sequencing is time consuming, relatively expensive, and would not provide an adequate clinical interpretation [14,16,17], highly reliable AI-integrated models will quickly analyze these high-dimensional data streams (refer to the training step, phase one), Figure 1A.

#### 2.7.2. Prediction and Pairing Step

Following the identification/interpretation of all aberrant pathways (in the first step), this information will be tested against a vast drug library to test/pair each target (gene, pathway, biomarker) with the potential drug to produce a target–drug combination and predict the response rate for each pair. This process will end with a diagram of the target response rates (%) hierarchy for each potential drug (A, B, and C) to produce a target–drug combination, as shown in Figure 1B. It is crucial to identify the drugs that will potentially interact with specific targets as many cancers require treatment with multiple agents and thus might potentiate the risk of side effects. At this stage, AI models can excavate numerous undesirable impacts through precise evaluation of potential interactions between multiple drugs (as in Step 2.1 Data collection and preprocessing) [78].

#### 2.7.3. Experimental Laboratory Validation Step (Pre-Clinical)

The results can be further examined experimentally in vitro and in vivo following the determination of a hierarchy of different drug response rates for each potential target–drug combination, as shown in Figure 1C. The validation step can be limited to the top five selections, for instance, rather than conducting experimental investigations on the whole obtained list. The standard protocol is to experimentally validate the transcriptomic results against the available agents in in vitro and in vivo examinations, which is costly and time consuming. By leveraging AI-integrated models, we can save both expense and time and offer a holistic approach to help recommend personalized treatment plans and choose the most effective treatment options.

#### 2.7.4. Introduction of Treatment to the Patient

Upon verification of the obtained data results and analysis from the previous steps, the best treatment option will be selected and discussed with the intended patient before delivery (Figure 1D).

## 3. Results and Expected Outcomes

**Figure 2:**
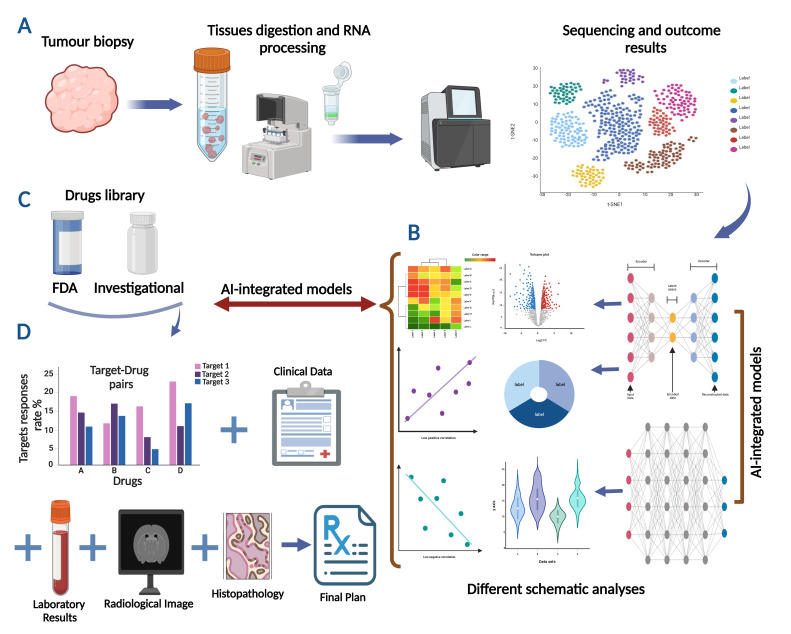
Schematic demonstration of AI-integrated models deployed to analyze the transcriptomic datasets and pair the obtained results with a compatible drug candidates. (**A**) Obtaining the tumour biopsy with subsequent tissue processing and RNA extraction followed by AI-based sequencing analysis. (**B**) Deep transcriptomic analysis using AI-integrated models resulting in different statistical and bioinformatics figures, such as identifying dysregulated pathways, detecting targeted genes, and recognizing molecular biomarkers. (**C**) Examining targets against comprehensive drug libraries to produce the best target–drug combination option. (**D**) Combining the AI-based target–drug pair results with other clinical and laboratory findings, imaging data, and histological pictures to reach a complete decision. (Created with BioRender.com, https://app.biorender.com/gallery, accessed on 6 August 2024).

1. Enhanced Data-Driven Insights:

Elucidation and dissection of the transcriptomic data point using the proposed cultivated AI-based technology will provide a profound elucidation and comprehensive analysis of the examined genomic profile. As demonstrated in Figure 2A,B, the gained analysis will cover various statistical and bioinformatics figures, such as identifying dysregulated pathways, detecting targeted genes, and recognizing molecular biomarkers relying on the comparison/normalization with the ground truth analysis status.

2. Improved Therapeutic Selection and Decision Making:

After the tumour is tested computationally against various treatment options according to the obtained genetic background, individual response patterns will be achieved through ongoing data analysis and model refinement. Thus, more accurate measurements of tumour response to different treatments and proactive treatment plan adjustments will lead to faster and more sustained improvements in results. Indeed, AI models such as DL and reinforcement learning (RL) models can predict the likely response rate of cancer cells to various treatment options by optimizing therapeutic plans based on patient-specific data and RNA expression profiles [79,80]. Moreover, AI-based algorithms can recommend several combination therapies to deliver a synergetic effect [79,80]. Finally, after producing the best target–drug combination option (Figure 2C,D), the AI model allows the incorporation of these results with other investigations, including clinical, genetic, and imaging data, laboratory findings, and histological pictures to draw a complete and clear framework of the patient’s medical status and therapeutic scheme [81].

3. Introduction of Personalized Care and Potential Cost-Effectiveness:

According to the previous results, AI-related algorithms, on the one hand, can provide plenty of alternative biological network interactions to distinguish the candidate targets based on various perspectives. On the other hand, DL models can compensate each other to manage high throughput data and denoise irrelevant biological features. This will provide a refined molecular explanation and accurately identify potential anticancer agents [36,82]. Validation of the selected target–drug combinations by conducting in vivo experimental verification will further confirm the results and tailor the treatment based on individual needs, maximizing treatment efficacy and minimizing trial-and-error interventions. Thus, eventually, this will lead to improved access and cost- and time-effectiveness.

## 4. Case Studies

Non-small-cell lung cancers (NSCLC) are the most common form of lung carcinoma, accounting for 85% of cases [83]. While surgical resection of the tumour remains the standard approach to NSCLC, specifically in the early stages of the disease, post-surgical trauma and complications are worrisome [84]. Furthermore, therapeutic resistance, metastases, and tumour relapse are other factors that contribute to the burden of the disease [85]. In the context of early diagnosis of NSCLC and cancer staging with the introduction of the best therapeutic plan, AI showed promising results [64,65]. This was accomplished by employing the DL algorithm, including the NLP software (version 1.0.34) in the CT film analysis, to guide physicians in accurately discriminating lung nodules and abnormalities [64,86]. Also, the DL model merged with PET and other imaging tools revealed improved predictions of different stages of lung adenocarcinoma and lung squamous cell carcinoma [87]. 

Furthermore, with the development of an automated system using a deep convolutional neural network (DCNN) in combination with generative adversarial networks, the GAN technique showed a significant enhancement in the efficiency of cytopathological diagnosis and classification of lung cancer cells [88]. This automated system demonstrated a precision rate of 85.3% during the classification of lung cancer tissues and a 4.3% higher accuracy rate than the standard protocol [88]. Notably, the ongoing advancement in AI technology and the engagement of AI-compressed data storage systems, CDSS, allow for choosing the best surgical approach for lung cancer patients, especially in metastatic conditions [89]. Additionally, the implantation of a cognitive computing system, IBM Watson for Oncology (WFO), based on integrating data analysis and image interpretation, provides a quick tool to extract relevant information from patients’ records and illuminate the best treatment choices [90]. Accordingly, Luo et al. have suggested a new filtering technique within a machine learning model to provide a rapid tool for personalizing lung cancer treatment by identifying molecular targets and drug candidates [91,92]. 

Neuro-oncology is another field where AI has significantly improved the management of patients with glioblastoma [73]. Glioblastoma is an aggressive brain tumour showing high morbidity and mortality rates due to the complexity of the disease diagnosis and treatment [93]. The grim progression in brain tumours, specifically glioblastomas, despite numerous clinical trials over the decade, has raised the urge to personalize treatment strategies that might potentially improve the response to therapy and alleviate side effects [73]. Indeed, numerous challenges need to be offset to obtain favourable outcomes; these include complex brain anatomy, tumour heterogeneity, difficult accessibility for acquiring imaging and biopsy sampling [94], lack of predictive biological biomarkers, and variability in response to various treatment modalities such as surgery, radiation, and chemotherapy [95]. Artificial intelligence is presented as a multidisciplinary tool in addressing many hindrances across different stages of glioblastoma management [73]. In this context, AI guides neuroradiologists in discriminating between tumour tissues and necrotic regions, accurately detecting the tumour site and segmentation, and offering precise quantitative measurements [96,97]. For example, AI models integrated with T2-weighted MRI [98] and DL-based algorithms can identify the exact tumour location and forecast the site of future relapse and the survival outcomes [99]. Furthermore, several DL-based models have been established, showing consistent performance in identifying tumour architecture, 3D segmentation, and removing noise and artifacts, including the 3D U-Net, DeepMedic, and V-Net models [100,101]. Moreover, DeepGlioma is an AI-based innovative technology that empowers radiologists to offer fast screening tool results (<90 s) by applying stimulated Ramen histology images [102]. 

By providing precise image analysis, AI assists neurosurgeons in drawing up a treatment plan and further guides them during the excision of the tumour, allowing them to identify the surgical margin and offering on-the-spot diagnosis and guidance, improving surgical outcomes [103]. Sturgeon is a recent DL-based system that uses sequencing methylation array data technology and provides a real-time, fast, and accurate classification of brain tumours in the surgical setting [104], thus informing the surgeons about the extent of the resection site and reducing postoperative complications [104]. Deep learning imaging signature (DLIS), combined with a pre-operative MRI scan, has been developed to present a non-invasive tool for detecting distinct mutations in low-grade glioma, providing a valuable diagnostic effect [102]. AI-based methods further contribute significantly to guiding the neuropathologist by delivering comprehensive analysis of the fresh pathological tissues, assisting tumour stratification, grading, and histomolecular classification [73]. Additionally, AI permits molecular pathologists to streamline the molecular land of the tumour involving mutation and variant data, methylation patterns, biomarker demarcation, and prediction of the therapeutic response [105]. Synergetic contributions of these various AI methods augment neuro-oncologists’ capabilities by enabling accurate screening and diagnosis, providing deeper insight into the disease pathology, facilitating personalized treatment, and visualizing prognosis and outcomes for brain tumour patients [73].

## 5. Discussion

The discovery of cancer treatment modalities continues to evolve, though cancer remains a critical health burden demonstrating high mortality and morbidity rates [1,2,3,4]. Because the pathogenesis of cancer is complex, most of the standard anticancer drugs have been developed based on experimental validation; hence, these therapies have unsought effects on normal tissues [36]. This has resulted in modifying the existing treatment pairwise with the invention of new strategies that have gradually met the current medical requirements [1]. Many struggles have been experienced in treating cancers, attributed to tumour nature diversity, genomic aberrations, and an increasing number of genetic mutations in each tumour [1,13]. The standard tool in diagnosis and classification in oncology is based on histopathological evaluation and the examination of the expression of specific markers or cell surface receptors [18,106,107]. However, intra-tumour heterogeneity represented a significant challenge that can interfere with the precise taxonomy and diagnosis of the tumour and hamper the accurate prediction of the drug candidate [18,106,107]. These challenges have ignited the desire to develop sophisticated, multifaceted, distinct genomic analysis technologies for routine cancer investigations [13]. Accordingly, extensive preclinical work and consolidated depiction of many cancers using high-throughput multi-omics technologies such as NGS have delivered new genomic cancer classifications for multiple cancer types and detected differentially expressed genes and biomarkers for diagnostic and therapeutic determination [12,13,17]. 

Applying genome-scale assays has significantly increased the usability of molecular databases to find a link between genotype and phenotype, recognition of novel biomarkers, stratification of tumours, and prediction of drug targets [82,108,109]. Moreover, this meticulous transcriptomic technology opened the gateway to introducing personalized/precision medicine into cancer care practice, i.e., one size does not fit all [18]. Personalized cancer therapy exhibited breakthrough avenues in resolving the obstacles, inhibiting tumour growth, and sparing normal cells from unwanted toxicities [1]. In this scenario, customized medicine has brought successful outcomes in drug selection for lung cancer patients with NSCLC based on the paradigms of their molecular profiles, specifically those with positive genetic alterations in EGFR, ALK, ROS-1, HER2, BRAF, MET, and RET genes [13,110]. Significantly, NSCLC, enriched for EGFR gene mutation (EGFR-activating mutations in exons 1821), has demonstrated enhanced sensitivity to EGFR tyrosine kinase inhibitors (TKIs, gefitinib) [111,112]. Additionally, EGFR-positive cancer patients revealed prolonged progression-free survival after receiving gefitinib treatment compared to patients who do not harbour EGFR gene mutations [113]. 

Nonetheless, the engagement of genome transcriptomic methods in aiding clinical diagnosis and treatment selection is limited and ascribed to many factors [15]. The first issue is that the large amounts of the generated genomic datasets must be managed by reliable, robust, comprehensive, and clinically applicable tools, which means the current standard statistical method cannot sufficiently meet these requirements [27]. Another issue is that technology is costly and time consuming, incompatible with the desirable short timeline required to make a treatment plan [13]. In this respect, AI-based models effectively contribute to extracting, interpreting, and analyzing cancer genomic figures more quickly [15,18]. Since cancer is a complicated disease with a heterogeneous genetic background requiring a multilayered data tool to explore its nature, AI, specifically the CNN-DL-based model, can play a significant role in cancer genomics [15,18]. AI technology simultaneously enables multitask learning with a suitable optimizer algorithm during training. Additionally, AI automatically provides a multimodal learning process through combining and analyzing different kinds of data [18]. These features empower AI to decipher the tumour genomic picture and recognize drug-candidate genes deeply [18]. 

Over the past decades, the integration of machine learning into risk stratification for genomic classifications of many malignancies has been acknowledged and has resulted in improved prognostication and treatment, such as Oncotype-Dx for breast cancer [5]. Machine learning has also been trained to predict metastasis after prostatectomy using the decipher score genomic classifier that relies on the expression of 22 biomarkers [5]. With the evolution of AI systems, the DL model has shown the capacity to interpret multimodal genetic data and tumour mutational burden, which assists in predicting the response to cancer immunotherapy [114]. Within this framework, the CNN-DL model, using specifically stained histopathology images, enables the characterization of various genetic alterations, including genetic and epigenetic heterogeneity, amplifications and deletions, and chromosomal variations for pan-cancers [115]. By estimating the expression profile of different genes in each tissue, the ExPecto approach offers a model for disease prediction and can explain the complex etiology of cancer based on input transcriptomic sequencing datasets [18,116]. Another AI-powered application is Splice AI, a 32-layer deep CNN that identifies abnormal splicing machinery from mRNA sequences for therapeutic targets [18,57,58]. Integrating AI models with CRISPR-based genome editing provides advanced methods for interpreting the patient’s genomic backgrounds and detecting mutations, alterations, and biomarkers associated with different illnesses, thus improving personalized medicine’s diagnostic/prediction capacities [51].

The applications of different AI models extend beyond the analysis and interpretation of cancer patients’ genomic datasets. The NLP and powerful language models have been adapted to extract and analyze scientific published works on oncology to generate more accurate evidence-based medicine [117]. Furthermore, adjusting drug dosage and predicting patients’ response to neoadjuvant chemotherapy [20] and immunotherapies [5] successfully revealed favourable patient outcomes in various cancers by employing DL models based on radiomic data and using automated RANO and RECIST assessments, respectively [5,20]. Considering cancer diagnosis, DL-based models exhibit high performance and efficiency as diagnostic tools because they can eliminate false positive results by fine-tuning and simplifying standard computer channels [20]. Around 20 FDA-approved AI-DL models are used explicitly for screening and diagnosis touch points in clinical oncology, including algorithms focusing on mammography and CT-based abnormality diagnosis [23]. Applying the Arterys Oncology DL CT/MRI scan to segment lung nodules and liver lesions with automated reporting has delivered satisfactory results [5]. Recent work has validated the efficacy and accuracy of a newly developed AI-based diagnostic system, the Liver Artificial Intelligence Diagnosis System (LiAIDS), in diagnosing focal liver abnormalities. LiAIDS was established by selecting retrospective and prospective clinical data from 12,610 patients from 18 hospitals. Additionally, LiAIDS can provide educational sources and instructional support for radiology trainees and hence serves as a valuable diagnostic tool in areas experiencing a shortage of radiology staff [118]. Moreover, non-invasive diagnosis of brain cancer (Cortechs NeuroQuant—MRI) [119], grading of prostate cancer (Quantib Prostate—MRI) [120], and the Hologic Genius AI Detection—DBT mammographic software (2.0) for detection of suspicious soft tissues and calcifications of the breast [121] are other modalities of DL clinical diagnostic models with high-qualified systems. 

Another clinical application that has demonstrated encouraging signs of DL model capability is the accurate classification of skin cancers utilizing digital photographs produced by CNN systems [22,122]. Interestingly, AI models have been customized for radiographic and histopathologic procedures to predict genomic alteration and classifications [123]. For example, AI models integrating machine learning and deep learning algorithms are deployed to guide different processes in the field of digital pathology [124]. These include big data collection and registration, screening, detection, segmentation, processing, speech recognition, and extracting features and information from medical digital images [125]. Applying this technology aids in precise diagnosis and analysis of different biomarkers and identifies prognostic and therapeutic responses based on the interpretation of pathological images [124]. Indeed, several reports have shown the efficacy of AI-based algorithms in identifying novel biomarkers and genetic alterations in different types of cancer by examining only hematoxylin and eosin (H&E)-stained pathological slides [115,126,127]. Introducing pathological AI models in neuro-oncology reveals excellent clinical implications for this technology in classifying and characterizing CNS tumours, specifically gliomas [127,128].

Artificial intelligence has unveiled another valuable contribution in the field of theranostics. Theranostics is a rapidly evolving field intending to enhance patient care under the precision/personalized medicine umbrella [129]. This division of science links qualified imaging tools with targeted therapy to assist in clinical decision-making and optimize treatment plans with efficient supervision [129]. The recent fruitful breakthroughs in AI enable this technology to offer innovative implementations in theranostics, producing a recognizable paradigm shift in personalized medicine [130]. The following example better demonstrates the integration between these two fields, AI and theranostics. In nuclear medicine, SPECT/CT or PET/CT scans can be used to follow the path of the radionuclide agent at different stages, i.e., pre-therapy and post-therapy, to obtain a complete picture of the treatment plan and outcomes, including the biodistribution and excretion of the radionuclides [131]. This tracking strategy provides deep insight into the effect of the treatment on diseased tissues and healthy ones. Yet, it is often considered impractical in a busy clinical setting limited by a specific time frame [131]. Notably, theranostics can combine pharmacokinetic information through imaging and correlate these results with dosimetry calculation [132]. In this regard, theranostics facilitates and simplifies this process by employing Hänscheid and prior-information methods [133,134]. The Hänscheid system calculates the absorbed dose following each round of radionuclide therapy in each SPECT/CT scan [133]. The prior-information session monitors the time–activity graph from multiple acquired scans after each cycle and applies this information to subsequent cycles [134]. The assimilation of AI models such as CNN and DL achieves further optimization and enrichment of this procedure [135]. Training CNN and DL models with dosimetry datasets and the acquired images at different time points results in the precise and comprehensive delimitation of the radionuclides’ pathway in the body in correlation with physiological and kinetic parameters [130]. Despite this sophisticated integration, the application of AI into different areas of theranostics merits extensive elaboration [130].

Combination therapeutic modalities targeting multiple molecular pathways can be superior to a single treatment regimen, thus ensuring synergetic power with minimum adverse effects on healthy tissues [7]. Such a promising strategy can provide favourable patient outcomes within the frame of personalized medicine. As in our proposal, leveraging AI models in clinical oncology to select the optimum target–drug combination candidates based on analyzing each patient’s unique molecular profile will deliver a firm foundation to enhance precision medicine for cancer patients. Despite substantial limitations in bridging the gap to clinical implementation of AI models in the oncology field, DL has paved the way for deciphering significant data streams as long as these pitfalls in the evolution process can be overcome [5].

## 6. Potential Challenges and Considerations

Several vital considerations should be carefully managed when deploying AI models in gene editing within healthcare practice:

- Data privacy and ethical consideration and compliance: Ensuring the privacy and security of patient data is imperative. Therefore, AI systems must adhere to security compliance and robust regulations like GDPR and HIPAA to guard sensitive material and avoid the exploitation of data by unauthorized individuals. Also, maintaining transparency with patients while delivering AI decision-guiding procedures and obtaining informed consent while avoiding undue influence in decision-making is paramount [51]. This should involve proper communication to address concerns about designing the plan, interventions, and service delivery [51].
- Bias in AI data curation: AI models can inherit biases present in training data. Training AI systems on diverse, representative, reliable, and updated datasets is crucial to avoid perpetuating existing biases. Furthermore, incorporating well-justified concepts of clinical validity and utility during the preliminary development and validation stage is critical for clinical translation and the successful introduction of AI into real-world cancer care [51].
- Accessibility and availability: Ensuring that AI-driven tools are accessible to all intended patients, including those in underserved communities, is essential for equitable healthcare [5].

## 7. Conclusions

Early diagnosis and accurate treatment of cancer are crucial to tackling the adverse consequences of the disease burden. Genomic sequencing modalities for different types of cancers have assisted in recognizing numerous therapeutic targets and understanding the molecular networks among other genes. Nonetheless, precise dissection of mass data remains a big challenge. Ongoing evolutions in computational algorithms have allowed AI to address multiple aspects of clinical oncology, from assembling and extracting patient data and handling large amounts of unstructured information to predicting the topmost disease management strategy. Moreover, AI-based approaches have demonstrated valuable guidance and played a pivotal role in conducting basic and clinical research regarding different medical conditions, offering innovative solutions to longstanding drawbacks. In this way, AI’s contribution is fundamental in empowering the precision, efficiency, and affordability of many healthcare amenities and scientific industries. Indeed, preclinical validation of some AI-based models has illuminated clinical trials, allowing us to examine the efficiency of cancer management further; nevertheless, enhanced interventions are needed to improve the performance and robustness of the AI-DL models [5,20,81]. Two main issues need to be addressed to facilitate the engagement of the DL model in clinical adoption. The first one is the extraction and interpretation of biological insights with superior reproducibility. The second issue is increasing the DL approach’s generalizability to validate and adapt to multi-institutional data repositories [136,137]. 

Given how dramatically the AI field is advancing and progressively engaging with the medical corpus, digitizing the healthcare system will be the scope of attention in the coming decade. With the exponential growth in patient data and characteristics, it is tempting to think that one could establish a comprehensive and dynamic technology to deliver precision oncology, enabling versatile and precise genetic modifications. Clinicians and scientists must prepare well for this upcoming era. Indeed, all healthcare providers must become familiar with the fundamental features of AI-related models to align with the various aspects of modern medicine. To preserve proper AI system validity, utility, accuracy, and clinical applicability, systematic amendment accompanied by regular structure updating and follow-up is vital. As knowledge grows, collaboration between AI and other disciplines, such as pathology, genomics, radiography, and electronic data streams, will continue to fill the gaps in the clinical and basic domains supplemented with influential applications for the betterment of human life.

## Data Availability

The original data contributions presented in the study are all included in the article; further inquiries can be directed to the corresponding author.

## Abbreviations

AI	Artificial Intelligence
PET	Positron Emission Tomography
SPECT	Single-photon Emission Computed Tomography
NGS	Next-Generation Sequencing
DL	Deep Learning
EGFR	Epidermal Growth Factor Receptor
NSCLC	Non-Small-Cell Lung Cancer
ML	Machine Learning
CNN	Convolutional Neural Network
XAI	Explainable Artificial Intelligence
FDA	Food and Drugs Administration
GEO	Gene Expression Omnibus
TCGA	The Cancer Genome Atlas
CCLE	Cancer Cell Line Encyclopedia
ENCODE	ENCyclopedia of DNA Elements
COSMIC	Catalogue of Somatic Mutations in Cancer
TTD	Therapeutic Targets Database
RF	Random Forest
SVM	Support Vector Machine
KNN	K Nearest Neighbour
NN	Neural Network
GBM	Gradient Boosting Machine
RNN	Recurrent Neural Networks
NLP	Natural Language Processing
DT	Decision Trees
GBDT	Gradient-Boosting Decision Trees
SNPs	Single Nucleotide Polymorphisms
BPSO-DT	Binary Particle Swarm Optimization Decision Tree
MHC	Major Histocompatibility Complex
MFO	Moth–Flame Optimizer
RL	Reinforcement Learning
DCNN	Deep Convolutional Neural Network
GAN	Generative Adversarial Networks
CDSS	AI-compressed Data Storage Systems
IBM—WFO	IBM Watson for Oncology
DLIS	Deep Learning Imaging Signature
LiAIDS	Liver Artificial Intelligence Diagnosis System
MRI	Magnetic Resonance Imaging
CT	Computed Tomography
